# The Real‐World Effect of 12 Months of Romosozumab Treatment on Patients With Osteoporosis With a High Risk of Fracture and Factors Predicting the Rate of Bone Mass Increase: A Multicenter Retrospective Study

**DOI:** 10.1002/jbm4.10637

**Published:** 2022-06-05

**Authors:** Hiroyuki Inose, Akane Ariga, Takayuki Motoyoshi, Kazuyuki Fukushima, Shoji Tomizawa, Tsuyoshi Kato, Kunihiko Takahashi, Toshitaka Yoshii, Atsushi Okawa

**Affiliations:** ^1^ Department of Orthopedic and Trauma Research Tokyo Medical and Dental University Tokyo Japan; ^2^ Department of Orthopedics Saku Central Hospital Advanced Care Center Nagano Japan; ^3^ Department of Orthopedics Tokyo Bay Urayasu Ichikawa Medical Center Chiba Japan; ^4^ Department of Orthopaedics Ome Municipal General Hospital Tokyo Japan; ^5^ Department of Biostatistics, M&D Data Science Center Tokyo Medical and Dental University Tokyo Japan; ^6^ Department of Orthopaedics, Graduate School Tokyo Medical and Dental University Tokyo Japan

**Keywords:** BONE MINERAL DENSITY, BONE TURNOVER MARKERS, FRACTURE, OSTEOPOROSIS, ROMOSOZUMAB

## Abstract

Excluding clinical trials, there is limited evidence on the effect of 12 months of romosozumab treatment on bone mineral density (BMD) increase in real‐world clinical practice because its use has only been approved recently. Thus, this study aimed to investigate the real‐world effect of 12 months of romosozumab treatment on BMD increase and identify factors that predict the rate of BMD increase after 12 months of romosozumab treatment. We retrospectively investigated 106 patients who completed a 12‐month romosozumab treatment for osteoporosis with a high risk of fractures at four hospitals from March 2020 to March 2022. The univariate and multiple regression analyses were performed to analyze the concurrent effects of various factors on the BMD increase after the 12‐month romosozumab treatment. After 1 year of treatment, the lumbar spine BMD increased by 14.6%, and femoral neck BMD increased by 5.1%. Univariate regression analysis found that male sex, high tartrate‐resistant acid phosphatase 5b (TRACP‐5b) value before romosozumab administration, absence of osteoporosis medications before romosozumab administration, and low baseline lumbar spine BMD were associated with the extent of lumbar spine BMD increase. Moreover, stepwise multiple regression analysis found that the TRACP‐5b value before romosozumab administration was a significant predictor of the rate of lumbar spine BMD increase after 1 year of romosozumab administration. In conclusion, our results demonstrated the effectiveness of the 12‐month romosozumab treatment for osteoporosis with a high risk of fractures and the TRACP‐5b value before romosozumab administration was a significant predictor of the rate of lumbar spine BMD increase after 1 year of romosozumab administration. Our findings could help establish more efficient treatment strategies for patients with osteoporosis at a high risk of fracture. © 2022 The Authors. *JBMR Plus* published by Wiley Periodicals LLC on behalf of American Society for Bone and Mineral Research.

## Introduction

The number of patients with osteoporosis is increasing with the aging of society,^(^ ^1^ ^)^ and, in Japan, 13 million people are affected by osteoporosis. Moreover, with osteoporosis, minor trauma can lead to fractures, which could subsequently lead to pain and even impairment in the patient's quality of life.^(^ ^2^, ^3^, ^4^, ^5^ ^)^ Recent advances in molecular biology have led to advances in the drug treatment of osteoporosis, including the development of bisphosphonates to inhibit bone resorption and teriparatide from promoting bone formation.^(^ ^6^ ^)^ However, bisphosphonates have the disadvantage of inhibiting bone formation as well as bone resorption, while teriparatide promotes bone resorption as well as bone formation.^(^ ^7^ ^)^ The anti‐sclerostin antibody, which was developed recently, is a drug that both promotes bone formation and inhibits bone resorption^(^ ^8^ ^)^ and has been reported to increase lumbar bone mineral density (BMD) by 10.7% to 16.9% in 12 months.^(^ ^9^, ^10^, ^11^, ^12^ ^)^ In March 2019, romosozumab was approved for use in Japan for osteoporosis with a high risk of fractures, ahead of other countries worldwide. Because the drug has only been approved recently, there are few reports on 12‐month romosozumab treatment for patients with osteoporosis in actual clinical practice.^(^ ^12^, ^13^, ^14^ ^)^ Although romosozumab has been found to be less effective in patients receiving osteoporosis medications, such as bisphosphonates, denosumab, or teriparatide, before receiving romosozumab,^(^ ^13^, ^14^, ^15^ ^)^ it is still difficult to predict in which patients it will be ineffective. Therefore, this multicenter retrospective study aimed to determine the real‐world effect of romosozumab on BMD and identify factors that predict the rate of BMD increase after 12 months of romosozumab treatment.

## Patients and Methods

### Study design

We retrospectively enrolled 123 patients who completed 12 months of romosozumab treatment for osteoporosis with a high risk of fractures at four hospitals (one academic medical center and three regional tertiary care hospitals) from March 2020 to March 2022. In this descriptive study, the inclusion criteria were patients who received romosozumab for 12 months for osteoporosis with a high risk of fractures. The following are the diagnostic criteria used by the Japanese Society of Bone Metabolism and the Japanese Osteoporosis Society to define osteoporosis with a high risk of fractures: (i) a BMD of ≤2.5 standard deviation (SD) with one or more fragility fractures; (ii) lumbar vertebral BMD of <3.3 SD; (iii) the presence of two or more existing vertebral fractures; and (iv) semiquantitative evaluation method results indicating the presence of grade 3 vertebral fractures.^(^ ^16^ ^)^

We performed dual‐energy X‐ray absorptiometry (DXA) to measure areal BMD at the spine (L_1_‐L_4_ total), total hip, and femoral neck before and after 12 doses of romosozumab. DXA equipment used in this study were Horizon (Hologic Inc., Bedford, MA, USA), Lunar iDXA (GE Healthcare Inc., Waukesha, WI, USA), and PRODIGY Fuga (GE Healthcare Inc.). The Japanese Society of Bone and Mineral Research and the Joint Review Committee of the Japanese Society for Osteoporosis reported the Japanese normative data for the percentages of the young adult mean at the lumbar spine and femoral neck based on the average values for people aged 20‐44 years and 20‐29 years, respectively.^(^ ^16^ ^)^ The least significant change (LSC) from baseline is 5.6% and 3.56% for the spine and total hip BMD, respectively.^(^ ^17^ ^)^ Among the 123 enrolled patients, 17 patients whose lumbar spine BMD was not measured before or after 12 doses of romosozumab were excluded. Because this study is a retrospective real‐world study, vitamin D and calcium supplementation was not mandatory and was left to the attending physician's discretion.

Blood samples were collected before romosozumab administration. To evaluate the patient's bone metabolism, we measured the following bone turnover markers: procollagen type 1 amino‐terminal propeptide (P1NP) to assess the bone formation and tartrate‐resistant acid phosphatase 5b (TRACP‐5b), a marker of osteoclast number, to indirectly assess bone resorption.^(^ ^18^, ^19^ ^)^ We also assessed serum total alkaline phosphatase (ALP) as a classical bone formation marker.

We also investigated other clinical factors, including age, sex, body mass index (BMI), serum creatinine, serum albumin, serum calcium, use of osteoporosis medications before romosozumab administration, vitamin D supplementation, calcium supplementation, times between first DXA scan to romosozumab initiation, and times between romosozumab initiation to second DXA scan.

### Ethics approval

This study was approved by the appropriate ethics committees and internal review boards at participating institutions and conducted in accordance with the recommendations of the Declaration of Helsinki. The opt‐out method was used to obtain the patient's consent.

### Statistical analysis

After assessing the data normality with the Shapiro‐Wilk test, we performed a paired *t* test to identify differences in the BMD between before and after 12 months of romosozumab treatment. The associations between baseline variables and change in the values (the difference between pretreatment and at the 12th month of treatment) of lumbar spine BMD were investigated in a multiple linear regression model using a forward‐backward stepwise procedure. First, the predictors associated with the dependent variable at a *p* value ≤0.25 in the univariate regression analysis were selected for inclusion in the model.^(^ ^20^, ^21^ ^)^ Second, a stepwise model selection procedure was carried out among these candidates along with the adjustment factors (age and sex). The model that attained the minimum Akaike's Information Corrected Criterion was selected as the appropriate model. Spearman correlation analysis was also performed. Because the change in lumbar spine BMD is greater than the change in femur BMD for the effect of romosozumab, the analysis in this study focused on factors that predict the change in lumbar spine BMD, not femoral BMD.

JMP version 14 (SAS Institute, Cary, NC, USA) was used for statistical analysis, and *p* values <0.05 were considered statistically significant. All data are presented as means + SD.

## Results

In total, 106 patients were included in this study. Table 1 shows the baseline characteristics of the patients.

**Table 1:** Baseline Characteristics of Patients

Characteristics	Value	Normal range
Age (years), mean ± SD	76.9 ± 7.9	
Sex, male, *n* (%)	9 (9)	
BMI (kg/m^2^), mean ± SD	20.7 ± 3.1	
Albumin (g/dL) (*n* = 104), mean ± SD	4.1 ± 0.5	4.0 to 5.0
Creatinine (mg/dL) (*n* = 104), mean ± SD	0.72 ± 0.20	Women 0.47 to 0.79 Men 0.61 to 1.04
ALP (U/L) (*n* = 86), mean ± SD	283.7 ± 122.3	115 to 359
TRACP‐5b (mU/dL) (*n* = 90), mean ± SD	544.2 ± 255.8	Women 120 to 420 Men 170 to 590
P1NP (ng/mL) (*n* = 69), mean ± SD	71.5 ± 46.6	Women 26.4 to 98.2 Men 18.1 to 74.1
Calcium (mg/dL) (*n* = 100), mean ± SD	9.4 ± 0.4	8.5 to 10.2
Presence of prior anti‐osteoporosis medication, *n* (%)	46 (43)	
With vitamin D supplementation during romosozumab treatment, *n* (%)	30 (28)	
With Calcium supplementation during romosozumab treatment, *n* (%)	5 (5)	
First DXA scan to romosozumab initiation (days), mean ± SD	48.4 ± 84.6	
Romosozumab initiation to second DXA scan (days), mean ± SD	363.3 ± 61.8	

ALP = alkaline phosphatase; BMI = body mass index; DXA = dual‐energy X‐ray absorptiometry; P1NP = procollagen type 1 amino‐terminal propeptide; TRACP‐5b = tartrate‐resistant acid phosphatase 5b.

At the end of the 12 months of romosozumab treatment, lumbar spine BMD increased by 14.6%, femoral neck BMD increased by 5.1%, and total femur BMD increased by 3.1%, all showing a significant increase compared to the pretreatment levels (Table 2).

**Table 2:** Average Bone Mineral Density (YAM) of Lumbar Spine, Femoral Neck, and Total Femur Before and 12 Months After Romosozumab Administration

Characteristic	Before (mean ± SD)	After 12 months (mean ± SD)	*p*
Mean percentage of YAM at lumbar spine (%) (*n* = 106)	65.8 ± 12.1	75.4 ± 13.9	<0.0001*
Mean percentage of YAM at femoral neck (%) (*n* = 94)	58.6 ± 13.0	61.6 ± 13.1	0.001*
Mean percentage of YAM at total femur (%) (*n* = 96)	64.6 ± 12.7	66.6 ± 12.7	0.001*

YAM = young adult mean.

* *p* < 0.05.

**Fig. 1:**
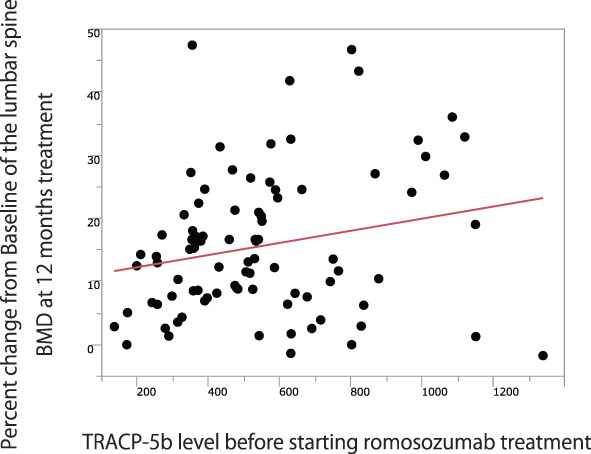
TRACP‐5b value before starting romosozumab treatment showed a trend toward positive correlation with percent change from baseline of the lumbar spine BMD at 12 months of treatment (*p* = 0.07).

Next, we performed a univariate regression analysis to examine the factors associated with the rate of increase in lumbar spine BMD. The results showed that male sex, high TRACP‐5b values before romosozumab administration, absence of osteoporosis medications prior to romosozumab administration, and low baseline lumbar spine BMD were significantly associated with the extent of lumbar spine BMD increase (Table 3). Furthermore, although not significant, there was a trend toward a positive correlation between the TRACP‐5b value before romosozumab administration and the extent of lumbar spine BMD increase at 12 months of treatment (Spearman's rank correlation coefficient = 0.20, *p* = 0.07) (Fig. 1).

**Table 3:** Univariate Regression Analysis. Association of Baseline Variables With Increase of Lumbar Spine Bone Mineral Density (YAM) From Before to After 12 Months

Characteristic	Estimation of partial regression coefficient	95% CI	*p*	Standardized β
Age (*n* = 106)	0.083	−0.177 to 0.344	0.53	0.062
Sex (*n* = 106)	−4.325	−7.922 to −0.728	0.02*	−0.228
BMI (*n* = 103)	−0.239	−0.917 to 0.438	0.49	−0.070
Albumin (*n* = 104)	−1.371	−5.810 to 3.067	0.54	−0.061
Creatinine (*n* = 104)	−0.453	−11.165 to 10.259	0.93	−0.008
ALP (*n* = 86)	0.003	−0.017 to 0.022	0.79	0.029
TRACP‐5b (*n* = 90)	0.010	0.001 to 0.019	0.04*	0.221
P1NP (*n* = 69)	0.047	−0.004 to 0.099	0.07	0.218
Ca (*n* = 100)	−1.811	−6.639 to 3.017	0.46	−0.075
Presence of prior anti‐osteoporosis medication	−2.261	−4.292 to −0.230	0.03*	−0.212
With vitamin D supplementation	−1.247	−3.520 to 1.026	0.28	−0.106
With calcium supplementation	−1.731	−6.577 to 3.114	0.48	−0.069
Baseline percentage of YAM at lumbar spine (*n* = 106)	−0.192	−0.358 to −0.026	0.02*	−0.219

ALP = alkaline phosphatase; BMI = body mass index; CI = confidence interval; P1NP = procollagen type 1 amino‐terminal propeptide; TRACP‐5b = tartrate‐resistant acid phosphatase 5b; YAM young adult mean.

* *p* < 0.05.

We then examined the independent predictors of the rate of increase in lumbar spine BMD after 1 year of romosozumab administration in a stepwise multiple regression analysis, adding age and sex as adjustment factors. Based on the univariate analysis, the dependent variable was defined as the increase in lumbar spine mineral density from pre‐treatment to the 12th month of romosozumab, and the candidate independent variables were TRACP‐5b, P1NP, use of osteoporosis medications before romosozumab administration, and baseline lumbar spine BMD with age and sex as adjustment factors. The results showed that the TRACP‐5b value before romosozumab administration was a significant predictor of the rate of increase in BMD after 12 months of romosozumab administration (Table 4). According to this prediction model, the following equation was obtained: the increase in lumbar spine mineral density from pretreatment to the 12th month of romosozumab = 8.776 + 0.080 × age + (male sex, 5.058; female sex, −5.058) + 0.009 × baseline TRACP‐5b value, *R* ^2^ = 0.11).

**Table 4:** Stepwise Multiple Regression Analysis: Independent Predictors of Increase of Lumbar Spine Bone Mineral Density (Young Adult Mean) From Before to After 12 Months

Factor	Estimation of partial regression coefficient	95% CI	*p*	Standardized β
TRACP‐5b	0.009	0.0001 to 0.018	0.048*	0.206
Sex	−5.058	−9.235 to −0.713	0.02*	−0.245
Age	0.080	−0.211 to 0.372	0.59	0.057

CI = confidence interval; TRACP‐5b = tartrate‐resistant acid phosphatase 5b.

* *p* < 0.05.

As for the fractures during the period of romosozumab treatment, three patients (2.8%) suffered major fragility fractures. Two patients had vertebral fractures, and one had a femoral neck fracture.

## Discussion

In this study, we found that 12 months of romosozumab treatment increased the lumbar spine BMD beyond LSC. Romosozumab also significantly increased the BMD of the total femur, although the increase did not exceed LSC. These BMD‐increasing effects are generally similar to those observed in previous reports.^(^ ^9^, ^10^, ^11^, ^12^, ^13^, ^14^ ^)^ Thus, these results suggest that romosozumab can be recommended for patients who fit the criteria for osteoporosis with a high risk of fracture.

The administration of osteoporosis medications before romosozumab administration was associated with an increase in BMD with romosozumab administration. Moreover, based on the regression coefficients, the administration of osteoporosis drugs before romosozumab administration had a negative effect on BMD increase with romosozumab. These results also support the results of previous studies.^(^ ^13^, ^15^ ^)^ Furthermore, based on the regression coefficients, patients with lower BMD before receiving romosozumab are more likely to have increased BMD with romosozumab treatment. This result also supports the results of previous studies.^(^ ^12^ ^)^ Taken together, romosozumab may be given first to patients with more severe osteoporosis to have a sufficient increase in BMD.

TRACP‐5b value before romosozumab treatment was an independent factor in predicting the rate of increase in lumbar spine BMD after 1 year of romosozumab treatment. Based on the regression coefficients, the higher the TRACP‐5b value before romosozumab administration, the stronger the effect of romosozumab administration in increasing BMD. These results are consistent with those reported by Tominaga and colleagues,^(^ ^12^ ^)^ who reported a positive correlation between baseline TRACP‐5b level and the percent change from baseline of the lumbar spine BMD at 12 months of romosozumab treatment. A new finding in our study is that TRACP‐5b was shown to be a predictor of increased lumbar spine BMD in univariate regression analysis and multivariate stepwise regression analysis after adjustment for age and sex. Several studies have reported that the P1NP value after 1 month of romosozumab treatment was a significant predictor of the rate of increase in BMD after 1 year of romosozumab treatment.^(^ ^13^, ^22^ ^)^ Unfortunately, we did not measure the P1NP values at 1 month; thus, we could not test whether the TRACP‐5b values before treatment or the P1NP values after 1 month of romosozumab treatment were more useful in predicting the rate of BMD increase in our data set. However, because the effect of the increase in bone formation appears early after romosozumab administration,^(^ ^9^ ^)^ it makes sense to conclude that the intensity of bone formation expressed by P1NP in the early postadministration phase is useful in predicting the rate of increase in BMD after 1 year of treatment, as reported by Ebina and colleagues.^(^ ^13^ ^)^ Conversely, the suppression of bone resorption marker β‐isomer of C‐terminal telopeptide of type I collagen by romosozumab administration, although weaker in degree than the initial increase in bone formation marker P1NP, continues throughout the administration period.^(^ ^9^ ^)^ Moreover, a study using bone biopsies confirmed that romosozumab also increased bone formation early and temporarily but persistently decreased bone resorption.^(^ ^23^ ^)^ The persistent bone resorption inhibitory action resulted in a decrease in bone turnover^(^ ^23^ ^)^; thus, the rate of increase in lumbar spine BMD may be predicted to some extent by the TRACP‐5b value before romosozumab administration. Based on the above data, it can be inferred that the higher the TRACP‐5b value before romosozumab administration and the higher the P1NP value after 1 month of treatment, the more effective the romosozumab administration. The results may also explain why the BMD‐increasing effect of romosozumab is most attenuated when denosumab, the most potent inhibitor of bone resorption at present, is administered.^(^ ^13^ ^)^ Consistent with the results of previous studies,^(^ ^12^, ^13^ ^)^ in our study, P1NP values before romosozumab administration tended to be associated with the rate of BMD increase, although not significantly (*p* = 0.07). From a clinical perspective, because teriparatide promotes both bone formation and resorption,^(^ ^24^ ^)^ patients with low levels of TRACP‐5b and P1NP before romosozumab administration may have a greater BMD increase if they receive teriparatide first and then romosozumab than if they receive romosozumab only. This treatment strategy should be investigated in future studies.

This study has several limitations. First, it is a retrospective analysis, and the sample size may have been slightly small. Second, missing data existed in this study. Particularly, P1NP could not be measured in 37 patients. Further prospective studies are needed to overcome these limitations.

In conclusion, 12 months of romosozumab treatment significantly increased the lumbar spine and hip BMD. Moreover, the TRACP‐5b value before romosozumab administration was an independent predictor of the rate of lumbar spine BMD increase after 12 months of romosozumab treatment. These findings could be useful in establishing more efficient treatment strategies for patients with osteoporosis at a high risk of fracture.

## Author Contributions

**HIROYUKI INOSE:** Conceptualization; formal analysis; investigation; project administration; writing – original draft; writing – review and editing. **Akane Ariga:** Investigation; writing – review and editing. **Takayuki Motoyoshi:** Investigation; writing – review and editing. **Kazuyuki Fukushima:** Investigation; writing – review and editing. **Shoji Tomizawa:** Investigation; writing – review and editing. **Tsuyoshi Kato:** Investigation; writing – review and editing. **Kunihiko Takahashi:** Formal analysis; investigation; writing – review and editing. **Toshitaka Yoshii:** Investigation; writing – review and editing. **Atsushi Okawa:** Project administration; supervision.

## Conflict of Interest

The authors declare no conflicts of interest associated with this manuscript.

### Peer Review

The peer review history for this article is available at https://publons.com/publon/10.1002/jbm4.10637.

## Data Availability

The data that support the findings of this study are available from the corresponding author upon reasonable request.
